# Mild Illness in Avian Influenza A(H7N9) Virus–Infected Poultry Worker, Huzhou, China, April 2013

**DOI:** 10.3201/eid1911.130717

**Published:** 2013-11

**Authors:** Huakun Lv, Jiankang Han, Peng Zhang, Ye Lu, Dong Wen, Jian Cai, Shelan Liu, Jimin Sun, Zhao Yu, Heng Zhang, Zhenyu Gong, Enfu Chen, Zhiping Chen

**Affiliations:** Zhejiang Provincial Center for Disease Prevention and Control, Hangzhou, China (H. Lv, Y. Lu, J. Cai, S. Liu, J. Sun, Z. Yu, Z. Gong, E. Chen, Z. Chen);; Huzhou Municipal Center for Disease Prevention and Control, Huzhou, China (J. Han, P. Zhang, D. Wen);; the 11th Chinese Field Epidemiology Training Program, Beijing, China (H. Zhang)

**Keywords:** avian influenza A(H7N9) virus, A(H7N9), avian influenza, influenza, viruses, mild case, poultry, poultry workers, wet market, poultry culling, China

## Abstract

During April 2013 in China, mild respiratory symptoms developed in 1/61 workers who had culled influenza A(H7N9) virus–infected poultry. Laboratory testing confirmed A(H7N9) infection in the worker and showed that the virus persisted longer in sputum than pharyngeal swab samples. Pharyngeal swab samples from the other workers were negative for A(H7N9) virus.

During March–May 2013, a respiratory disease caused by avian influenza A(H7N9) virus was identified among humans in China (*1*–*6*). Most infected persons were >60 years of age, and most cases were severe and involved serious complications, including death (*1*). Few children and adults have been reported with mild illness caused by influenza A(H7N9) virus infection (*7*,*8*). After an epidemiologic link was reported between exposure to poultry and confirmed influenza A(H7N9) cases (*9*–*11*), local governments closed contaminated wholesale wet markets (large markets where live chickens were sold to vendors) and assigned government office workers to assist in a temporary poultry culling campaign.

The largest number of confirmed cases was reported in Zhejiang Province, where 46 cases and 11 deaths occurred (data from the Chinese Disease Surveillance Information Report and Management System; as of July 20, 2013). Of the 46 cases, 12 were reported from Huzhou city, where the environment of a wholesale wet market was contaminated by influenza A(H7N9) virus (*9*). Approximately 25,000 live chickens were processed daily at this market, and on April 8, 2013, the Huzhou city government launched their campaign to close the market and slaughter the remaining poultry.

Sixty-one government workers participated for 3 hours in the culling campaign. The workers wore individual personal protective equipment, including protective clothing, ordinary disposable masks, and latex gloves; neither goggles nor face shields were worn (Figure 1). During the culling process, workers disarticulated chickens’ necks and placed the dead birds in individual sacks.

**Figure 1:**
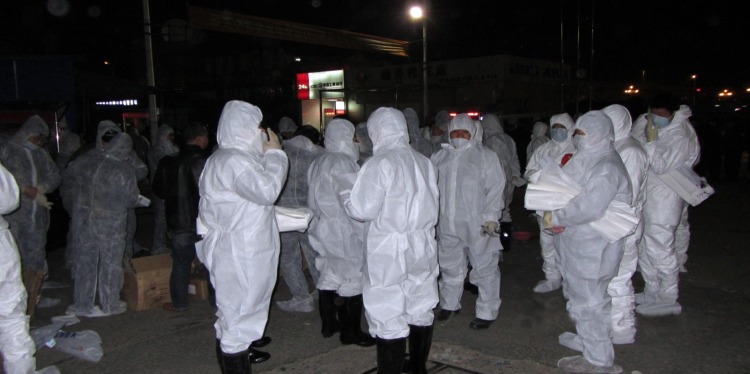
Personal protective equipment worn by government workers assigned to cull poultry at a wet market in Huzhou city, Zhejiang Province, China, April 8, 2013. The protective clothing included ordinary disposable masks and latex gloves but not goggles or face shields.

Avian influenza A(H7N9) infection was subsequently confirmed in 1 of the 61 workers. We conducted an epidemiologic investigation and clinical review of the confirmed case. In addition, we administered questionnaires to the 60 co-workers and obtained pharyngeal swab samples from them to test for influenza A(H7N9) virus.

## Case Report

The male patient was a 41-year-old administrative manager in a sub-district government office in Huzhou city. The patient had been a chronic smoker, but discontinued smoking 4 years earlier because of chronic pharyngolaryngitis. He did not report any other underlying medical conditions, including hypertension and diabetes.

The patient’s only contact with poultry during the 10 days before symptom onset occurred on April 8, when he participated in the campaign to cull poultry. Five days later, on April 13, the patient’s eyes were swollen, but there was no tearing or discharge. Midday on April 14, the patient experienced low-grade fever (self-reported axillary temperature 37.8°C), dry throat, cough with a small amount of white sputum, weakness, and muscle soreness. Later that afternoon, he visited the outpatient clinic of Huzhou First People’s Hospital. Clinical records were not available, however, at that visit, the patient was told his temperature was normal and that he probably had a cold, and he was sent home without medications.

The next morning, April 15, the patient returned to the clinic for medical evaluation and was found to have an oral temperature of 37.5°C, normal auscultation of the heart and lungs, leukocyte count of 5.3 × 10^9^ cells/L (reference range 4.0–10.0 × 10^9^ cells/L), neutrophil count of 3.25 × 10^9^ cells/L (reference range 2.0–7.0 × 10^9^ cells/L), and C-reactive protein level of 2.29 mg/L (reference range 0–4.0 mg/L). The patient was sent home without treatment, but later that afternoon, he returned to the clinic. At that third visit, a pharyngeal swab sample was collected and submitted to the Huzhou Municipal Center for Disease Prevention and Control for testing by real-time reverse transcription PCR (rRT-PCR) (*12*); the sample was found to be positive for influenza A(H7N9) virus. That same evening, leftover sample was confirmed positive for influenza A(H7N9) virus by a provincial reference laboratory.

During the early morning hours of April 16, immediately after laboratory confirmation of influenza A(H7N9) infection, the patient was transferred to a hospital designated for the care of persons infected with the virus. The patient’s oral temperature was recorded twice a day: his temperature was 37.6°C on the first morning of hospitalization and <37.5°C thereafter. Routine blood tests showed that the patient’s maximum C-reactive protein level (4.19 mg/L on day 4 of hospitalization) was slightly elevated, his leukocyte and lymphocyte counts were normal, his lactate dehydrogenase level was normal, and his neutrophil count ranged from normal to slightly below normal (Table 1). A cardiac ultrasound examination revealed no abnormalities in the heart, and results of an abdominal ultrasound of the liver, kidneys, and spleen was also unremarkable. Computer tomographic scans of the chests on April 16 and 18, showed an old lesion in the lung that seemed unrelated to the acute infection.

**Table 1:** Blood test results on hospitalization days 1–4 for a man infected with avian influenza A(H7N9) virus, Huzhou city, Zhejiang Province, China, 2013*

Index	April 16			April 17		April 18			April 19	Reference range
	Morning	Afternoon		Morning		Morning	Afternoon		Morning	
Leukocyte count (10^9^ cells/L)	7.0	5.9		4.9		4.4	4.3		4.0	4–10
Neutrophil count (10^9^ cells/L)	5.6	3.9		3.0		1.8	2.4		1.8	2–7
Lymphocyte count (10^9^ cells/L)	0.6	1.5		1.6		2.2	1.5		1.9	0.8–4.0
C-reactive protein (mg/L)	ND	ND		ND		3.4	2.9		4.19	0–4.0
Lactate dehydrogenase (IU/L)	183.0	ND		156.0		161.0	ND		142.0	106–211

*ND, not determined.

The patient was administered oseltamivir (75 mg 2×/day) on hospitalization days 1–6; noninvasive ventilation and symptomatic and supportive treatment were also administered daily. Each day while the patient was hospitalized, pharyngeal swab samples and sputum samples were collected and tested for the presence of influenza A(H7N9) virus by rRT-PCR. All pharyngeal swab samples, except the 1 obtained the day before hospitalization, were negative for the virus. Sputum samples were influenza A(H7N9) virus–positive on hospitalization days 1–3 and converted to virus-negative on hospitalization day 4.

**Figure 2:**
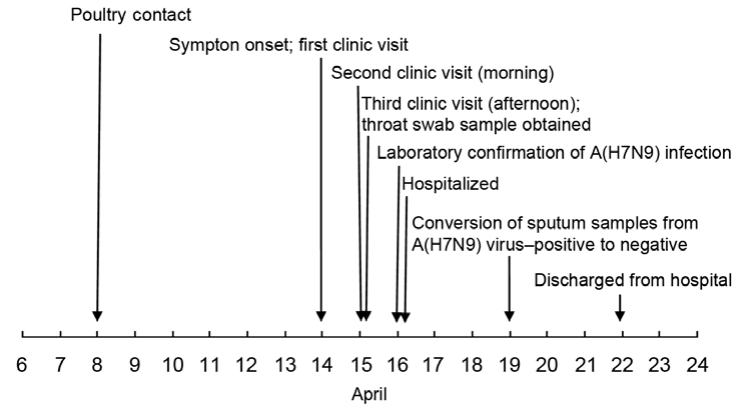
Timeline from exposure to avian influenza A(H7N9) virus to symptom onset, medical examination, hospitalization, laboratory confirmation of infection, and hospital discharge for a patient whose only contact with poultry occurred when he helped cull poultry at a wet market in Huzhou city, Zhejiang Province, China, April 2013.

As of April 22, 2013, the patient had made a good recovery and was discharged from the hospital. Figure 2 shows the timeline of events, from exposure to hospital discharge, for the patient.

On April 16, 2013, we administered a questionnaire to, recorded oral temperatures for, and obtained pharyngeal swab samples from the patient’s 60 co-workers (Table 2). None of the workers had fever or other signs or symptoms of infection at the time of screening, but 13 of the 60 reported transient symptoms during April 9–16. All pharyngeal swab samples from these workers were negative for influenza A(H7N9) virus by rRT-PCR.

**Table 2:** Demographic and screening information for workers who culled avian influenza A(H7N9)–infected chickens at a live market, Huzhou city, Zhejiang Province, China, April 16, 2013*

Variable	No. workers, N = 60
Age, median y (range)	36 (22–57)
Male sex	60
Occupation	
Government worker	10
Police officer	21
Code enforcement officer	29
Transient symptoms, April 8–16	
Mild cough	6
Mild cold	1
Upper respiratory infection†	1
Itchy throat	2
Dizziness (feeble)	2
Muscular soreness, diarrhea	1
Oral temperature <37.5°C	60
Pharyngeal swab sample negative for A(H7N9) virus	60

*A co-worker not listed here became ill 6 days after culling, and on April 16, the day of this survey, he was confirmed to be infected with influenza A(H7N9) virus. †This worker’s illness was diagnosed by a clinician on April 10, 2013.

## Conclusions

Our epidemiologic investigation and clinical review showed that mild upper respiratory symptoms developed in a man 6 days after he had contact with influenza A(H7N9) virus–infected poultry. We found that sputum samples from this patient remained positive for A(H7N9) virus longer than pharyngeal swab samples. This finding is in agreement with those of Chen et al. (*10*) and Lo et al. (*13*). Thus, it is a limitation of our screening of the patient’s 60 coworkers that we did not collect sputum specimens. Because of this, we may have missed identifying other mild infections among the workers who culled poultry.

The patient in this report is 1 of only a few adults with mild respiratory symptoms who have been confirmed to be infected with avian influenza A(H7N9) virus (*8*). Our investigation strongly suggests that he became infected with the virus after working for 3 hours as poultry culler in a contaminated wet market. Future investigations of persons exposed to influenza A(H7N9)–infected poultry may consider testing for the virus in sputum samples rather than throat swab samples.
